# Multiple Bioactivities of Traditional Medicinal Herbs for Treatment of Neurodegenerative Diseases

**DOI:** 10.1155/2018/3075458

**Published:** 2018-04-24

**Authors:** Gunhyuk Park, Irawan W. Kusuma, Yong-ung Kim

**Affiliations:** ^1^Ektos Industries Co., Ltd., Daejeon, Republic of Korea; ^2^The K-Herb Research Center, Korea Institute of Oriental Medicine, Daejeon, Republic of Korea; ^3^Institute of Human-Environment Interface Biology, Seoul National University College of Medicine, Seoul 03080, Republic of Korea; ^4^Department of Dermatology, Seoul National University College of Medicine, Seoul 03080, Republic of Korea; ^5^Forest Products Chemistry Laboratory, Faculty of Forestry, Mulawarman University, Samarinda, Indonesia; ^6^Department of Pharmaceutical Engineering, College of Biomedical Science, Daegu Haany University, Gyeongsan, Republic of Korea

Neurodegenerative diseases are debilitating and incurable conditions of the central nervous system. Characterized by neuronal dysfunction, they are often associated with atrophy of the affected nervous system structures. Age-related dementia constitutes a major subset of neurodegenerative diseases. Alzheimer's disease (AD) is the most prevalent clinical form of dementia in aging populations: 43% of individuals ≥85 years of age are thought to have AD in the United States. Parkinson's disease (PD), another prevalent neurodegenerative disease among the elderly, affects 1%–3% of the population over 60 years of age. By 2050, the United Nations predicts that, worldwide, 400 million individuals ≥80 years of age will have a neurodegenerative disease. Owing to the financial, societal, and personal impact of these diseases, the etiologies, prevention, and treatment have become the major focus of basic and clinical research. Associations between neurodegenerative diseases and genetic factors are reported; however, variations exist within populations for one disease state. AD is also linked to mutations in a specific gene; however, the downstream effects of its protein product on symptoms, including dementia, are not fully understood. Multiple signaling pathways govern the molecular basis of the effects of genetic variation, lifestyle, and environmental factors, including trauma and infection, in neurodegenerative diseases. The neuropathological hallmarks of dementia include *β*-amyloid plaques and neurofibrillary tangles in AD and Lewy body inclusions in PD. However, although misfolded protein aggregation clearly contributes to neurodegenerative disease, it is only a signature of neuronal damage and additional causative factors remain to be identified. Inflammation and nitric oxide signaling are active areas of investigation. The effects of these, and other key factors, on the transcriptional regulation and initiation of apoptosis and neurotoxicity continue to be explored. The pathogenesis of neurodegenerative diseases remains unclear; the present “one-drug, one-target” paradigm for neurodegenerative diseases appears clinically unsuccessful. The pathological effects of neurodegenerative diseases result from altered activity in multiple pathways. Thus, new pharmacological therapeutic strategies, using natural compounds or medicinal herbal extracts comprising multiple compounds, are specifically designed to act on multiple neural and biochemical targets.

In this special issue, we invited investigators to contribute original research articles and reviews that will help understand the basic mechanisms and development of multiple bioactivities and potential herbal medicinal strategies in the treatment of neurodegenerative diseases. Seven studies regarding the multiple bioactivities of traditional medicinal herbs in treating neurodegenerative diseases are included.

C.-Y. Kim et al. reported the therapeutic effects and mechanisms of 6-gingerol, a pungent ingredient of ginger, on scopolamine-induced learning and memory impairments in C57BL/6 mice. They suggested that 6-gingerol may result from the elevated protein expression of brain-derived neurotrophic factor mediated via the activation of the protein kinase B (Akt)/Akt- and cyclic adenosine monophosphate-response element binding protein signaling pathway; thus, this may be a candidate for the treatment of AD.

Y. Yang et al. demonstrated the anti-tau effects of Fuzheng Quxie Decoction (FQD), a Chinese herbal complex clinical formula, on learning and memory impairments in senescence-accelerated mice prone-8. Their results suggested that the effects of FQD, which may be a candidate for the treatment of AD, are mediated through the inhibition of hippocampal tau hyperphosphorylation via N-methyl-D-aspartate receptor/protein phosphatase 2A-associated proteins.

M. Liu et al. demonstrated the anti-amyloid precursor protein (APP) cleavage and amyloid *β* effects of Huannao Yicong Decoction (HYD) extract, a Chinese herbal formula, on the spatial learning and memory ability of transgenic APP/PS1 mice. Their study suggested that HYD extract clearly inhibited *γ*-secretase activity resulting from the inhibition of cyclic adenosine monophosphate-response elements activity and subsequent downregulation of presenilin enhancer 2 expressions.

Y. J. Kim and and H.-S. Lim et al. reported the effect and possible mechanism of Jodeungsan (JDS), a traditional herbal formula, on lipopolysaccharide or H_2_O_2_-induced neurotoxicity in hippocampal neurons and microglia and suggested that JDS extracts might suppress tumor necrosis factor-*α* and interleukin-6 production, supporting the antineuroinflammatory activity.

G.-Y. Lee et al. demonstrated the antioxidative effects of *α*-pinene on scopolamine-induced learning and memory impairments in C57BL/6 mice and suggested that the effects may be due to elevated expression of choline acetyltransferase and antioxidant enzymes, mediated via activation of the NF-E2-related factor 2 (Nrf2) pathway. Thus, *α*-pinene may be a candidate for the treatment of AD.

N.-Y. Chen et al. demonstrated the antiaging effects of* Fructus Cannabis* (EFC) from hempseed on D-galactose-induced learning and memory impairments. Their results suggested that the effect of EFC was mediated via inhibition of glia activation, tau phosphorylation, and the expression of presenilin 1 via reactive oxygen species generation, which would be useful to relieve age-related memory impairments.

L. Wu et al. reported the antioxidative effects of salidroside, a major component extracted from* Rhodiola rosea* L., on MPP^+^-induced neurotoxicity. Their study suggested that the effects of salidroside were mediated via upregulation of the PD protein 7/Nrf2-antioxidant pathway and associated antioxidant enzymes; therefore, it may be a candidate for the treatment of PD and associated neurodegenerative diseases.

L. E. M. Brandão et al. reported the antioxidative effects of* Passiflora cincinnata* Masters (PAS), a native Brazilian species of passionflower, on reserpine-induced neurotoxicity; they suggested that PAS delays the development of motor impairment and prevents dopaminergic neuronal loss and thus may be a potential candidate for PD treatment.

J. Chen et al. reviewed the potential of the fruits of* Ziziphus jujuba*, known as jujube or Chinese date, and assessed their phytochemical, ethnobotanical, phytotherapeutical, and pharmacological properties, combining a traditional perspective with recent neurological and scientific observations. Their efforts will assist future researchers in studying the dynamic ethnobotanical and phytotherapeutical roles of* Ziziphus jujuba* and develop novel ideas regarding the therapeutic roles of health supplements in the prevention and/or treatment of neurological diseases.

We hope that the readers will be interested in neurodegenerative diseases and that this special issue will attract the interest of the scientific community, thereby supporting further investigations that lead to the discovery of novel strategies and therapeutic medicinal herbs for use in the field of neurodegenerative diseases.

